# Diabetes Management Using Modern Information and Communication Technologies and New Care Models

**DOI:** 10.2196/ijmr.2193

**Published:** 2012-10-04

**Authors:** Emmanouil G Spanakis, Franco Chiarugi, Angelina Kouroubali, Stephan Spat, Peter Beck, Stefan Asanin, Peter Rosengren, Tamas Gergely, Jesper Thestrup

**Affiliations:** 1 Computational Medicine Laboratory (CML)Institute of Computer Science (ICS)Foundation for Research and Technology - Hellas (FORTH)Heraklion, CreteGreece; 2 JOANNEUM RESEARCHForschungsgesellschaft mbHGrazAustria; 3 CNet Svenska ABDanderydSweden; 4 Applied Logic LaboratoryBudapestHungary; 5 In-JeT ApSBirkeroedDenmark

**Keywords:** Medical Information Systems, Medical Expert Systems, Biomedical Engineering, Biomedical Informatics, Biomedical Computing, Telemedicine, Diabetes

## Abstract

**Background:**

Diabetes, a metabolic disorder, has reached epidemic proportions in developed countries. The disease has two main forms: type 1 and type 2. Disease management entails administration of insulin in combination with careful blood glucose monitoring (type 1) or involves the adjustment of diet and exercise level, the use of oral anti-diabetic drugs, and insulin administration to control blood sugar (type 2).

**Objective:**

State-of-the-art technologies have the potential to assist healthcare professionals, patients, and informal carers to better manage diabetes insulin therapy, help patients understand their disease, support self-management, and provide a safe environment by monitoring adverse and potentially life-threatening situations with appropriate crisis management.

**Methods:**

New care models incorporating advanced information and communication technologies have the potential to provide service platforms able to improve health care, personalization, inclusion, and empowerment of the patient, and to support diverse user preferences and needs in different countries. The REACTION project proposes to create a service-oriented architectural platform based on numerous individual services and implementing novel care models that can be deployed in different settings to perform patient monitoring, distributed decision support, health care workflow management, and clinical feedback provision.

**Results:**

This paper presents the work performed in the context of the REACTION project focusing on the development of a health care service platform able to support diabetes management in different healthcare regimes, through clinical applications, such as monitoring of vital signs, feedback provision to the point of care, integrative risk assessment, and event and alarm handling. While moving towards the full implementation of the platform, three major areas of research and development have been identified and consequently approached: the first one is related to the glucose sensor technology and wearability, the second is related to the platform architecture, and the third to the implementation of the end-user services. The Glucose Management System, already developed within the REACTION project, is able to monitor a range of parameters from various sources including glucose levels, nutritional intakes, administered drugs, and patient’s insulin sensitivity, offering decision support for insulin dosing to professional caregivers on a mobile tablet platform that fulfills the need of the users and supports medical workflow procedures in compliance with the Medical Device Directive requirements.

**Conclusions:**

Good control of diabetes, as well as increased emphasis on control of lifestyle factors, may reduce the risk profile of most complications and contribute to health improvement. The REACTION project aims to respond to these challenges by providing integrated, professional, management, and therapy services to diabetic patients in different health care regimes across Europe in an interoperable communication platform.

## Introduction

Diabetes mellitus is a metabolic disorder characterized by hyperglycemia (high blood sugar) resulting from defects in the production or response to insulin [1]. The disease has two main forms: type 1 and type 2. Type 1 disease is characterized by diminished insulin production resulting from the loss of beta cells in the pancreatic islets of Langerhans, in most cases caused by immune-mediated cell destruction. Disease management entails administration of insulin in combination with careful blood glucose monitoring. Type 2 diabetes patients, mostly over 50 years old (although more and more young people develop type 2 diabetes) with additional health problems (eg, cardiovascular disease), in the early stages are often characterized by high plasmatic insulin concentration. In the fasting state, the basal insulin secretion rate increases as a function of the progressive insulin resistance [2]. In later stages of type 2 diabetes, beta cells are unable to produce enough insulin, and then type 2 becomes more similar to type 1 [3]. Management principally involves the adjustment of diet and exercise level and the use of oral anti-diabetic drugs and, eventually, insulin to control blood sugar. Type 2 diabetes is one of the faster growing chronic conditions in the developed world and is closely linked to the emerging epidemic of obesity and unhealthy lifestyles, which are among the main causes of preventable health problems [4].

### Glucose Control in Diabetes Therapy for Insulin-Dependent Patients

Blood glucose is typically measured in a drop of capillary blood using a disposable dry chemical strip and reader device, an uncomfortable and slow process. Tight glucose control requires almost continuous measurements, and different sensors for continuous blood glucose measurement have been under development in the last two decades. Minimally invasive sensors able to measure glucose in interstitial fluid, more suitable for self-monitoring, have also been developed. To date, however, none of these has delivered a level of performance sufficient for use in routine glucose monitoring. Robust, clinically acceptable devices are expected to become widely available in the near future [5].

Although several guidelines for treatment regimen for management of diabetes have been defined [6-8], no adequate implementations of treatment regimen have been found for the establishment of glycemic control of hospitalized patients [9,10]. Studies have shown that frequency of hyperglycemia in surgical intensive care units can amount to 50-70% of all admitted patients [11].

Comprehensive management of diabetes has to be performed both in hospitals and outside health care premises. Based on the emerging clinical evidence from several clinical studies, there are worldwide increasing efforts to establish tight glycemic control in critically ill and hospitalized patients [12-14]. Achieving the goal of tight glycemic targets requires extensive nursing efforts, including frequent glucose monitoring, training of patients and carers to handle control algorithms, guidelines with intuitive decision making, and most importantly, additional responsibility to prevent hypoglycemic episodes. Traditional diabetes therapies for insulin-dependent patients try to achieve normal glycemia by administrating synthetic insulin to control patient’s blood sugar level. Given that insulin cannot be taken orally, patients must turn to special type of devices to administer insulin.

### Insulin Delivery Devices for Diabetes Therapy

The insulin needs of the body are covered with basal insulin, representing the background insulin (taking into account the daily activity level), which is normally secreted by the pancreas irrespectively of meals, and bolus insulin, representing the extra insulin necessary for balancing the glucose taken with food, which depends on the size of the meal.

During recent years, a number of insulin delivery systems have become available making insulin administration much easier. Many factors influence the choice of the appropriate device for each patient including patient conformance and self-care capacity. Most commonly, insulin is delivered using a needle injection or a syringe, an insulin pen, or an insulin pump.

A syringe is the simplest device used for the injection of insulin, where the patients typically use disposable units to prevent contamination and infection. An insulin pen is an injection device the size of a pen that includes a needle and holds a vial of insulin. There are two different types of insulin pens: a) durable with replaceable insulin cartridges; and b) disposable prefilled with insulin. The advantage of insulin pens over insulin syringes is that they are much more convenient and easier to transport and use than traditional vial and syringe. They can repeatedly administer more accurate dosages (especially for patients with visual or motor skills impairments). Insulin pens usually cause less pain to the patient. One of the disadvantages of insulin pens over insulin syringes is that, unlike traditional syringes, two different insulin solutions cannot be mixed in an insulin pen. Also, using pens needles is usually more expensive than using the traditional vial and syringe method. A major disadvantage of insulin injection devices is that they are designed to administer insulin only in large boluses, which can cause peaks and valleys in the blood sugar levels in patients.

A solution to this problem of insulin shots is provided when using insulin pumps. These devices, which are worn outside of the patient’s body, can be programmed to deliver a steady supply of insulin throughout the day at a basal rate and/or programmed to deliver larger boluses of insulin before or after meals. With a pump, continuous doses of background insulin are delivered to support the body’s needs between meals, and with a button press it is possible to obtain an “on demand” dose of insulin (a bolus) to cover instant needs. Each basal rate can be variable since the pump can be programmed to deliver different basal rates throughout the day. The main advantage of insulin pumps is that the individuals do not need to take multiple injections every day, allowing them to continue with their daily actions without any problem. The main disadvantage of insulin pumps, apart from the obvious discomfort that a person might feel wearing it, is the cost of the pump and its maintenance, which can get very high. Furthermore, patient’s activity could force the infusion set of the pump to slide off and stop the necessary delivery of insulin to the patient’s body. Therefore, it is very important for patients to monitor their blood glucose levels much more frequently making sure the pump is working correctly and avoiding risks of ketoacidosis.

## Methods

### The Need for New Care Models

Health care practice supported by electronic processes and communication (eHealth) provides new possibilities for revising central parts of the established care models for chronic diseases. Planning new care models for the future involving eHealth is highly complex and involves a number of different factors that influence the opinions and attitudes of the participants in health care systems and their ability to carry out changes that are needed to implement new care models [15].

**Figure 1:**
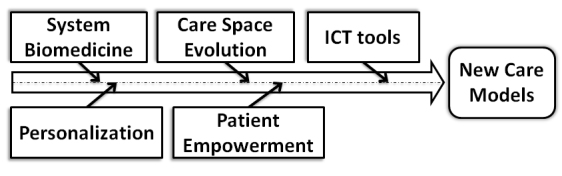
Significant factors influencing the new care models.

Several prediction studies have been published by health institutions as well as private observers. These studies differ in their approach and in the aspects they consider. Some studies emphasize technological aspect [16,17]; other focus on health care policies [18,19]. A study from the Australian Centre for Health Research focuses on the aspects of health services [20]. Some common elements can be extracted from these approaches, and the most significant factors for the formation of new care models are presented in Figure 1 [21,22].

Chronic diseases share three important features: i) acute and chronic phases alternate through time; ii) adequate diagnosis and state monitoring require multilevel system biomedical characterization; and iii) disease progress is significantly influenced by patient’s behavior. Care for chronic diseases therefore must be: i) continuous between visits and hospitalization periods; ii) proactive and predictive; iii) provided by medical personnel as professional care and by patients as self-management; iv) influencing the patient’s lifestyle; and v) dynamic, since all participants should learn and adapt during the care process.

Important aspects of chronic disease management are personalization, inclusion, and patient empowerment. These aspects are closely connected to care space evolution, ie, how the physical or virtual spaces of care change. In this regard, Information and Communication Technology (ICT) tools and biomedical technology act as enablers of mobile and remote health solutions and thus have an important role in care space evolution models.

Recent results in eHealth explicitly split the care space into two interrelated spaces, the activity and the information space. Activities are implemented in a distributive way while the necessary data and information come from an integrated and unified information space. A shared access is available from different activity spaces into the information space. There are activity spaces of the patients, the care teams, and the care provider, as well as a virtual one of the intelligent agents. The information space and the activity spaces together generate the integrated care space, which promotes the effective application of info-communication technologies and unites the participants into a virtual organization [21,22].

This model of care allows patient mobility to decouple the actual activity space from the traditional physical care space of a professional health care system, placing emphasis on the sound development of the care space. New health care methods can create a growing volume of data on the one hand, and an ever growing demand for information, on the other hand. The data growth is connected with the development of diagnostic and monitoring methods and tools. The information demand is connected with new decision making methods and with modeling methods required for a better implementation of the necessary actions.

The separation of care spaces in chronic disease management allows streamlining of the roles of each stakeholder while at the same time providing them with tailored information for each role. Also, if managed correctly, it provides great opportunities for improved care at the point of need as well as organizational streamlining and potentials for cost savings. 

In this case, in order to define new care models in the context presented, a challenge to overcome is the risk of the patient being isolated or alienated. If visits by care givers or family members are completely substituted with monitoring tools, patients can become isolated even in the most populated areas. It is imperative that a delicate balance is struck between closing the digital divide and closing patients in a virtual prison. Thus, inclusion enhancing methods must be incorporated in the new care models at all levels [23].

### Advanced ICT Solutions in Diabetes Management: The REACTION Project

Due to demographic changes, European health care systems face two serious challenges: health care delivery may become inadequate for the perceived needs of the citizens or the cost may spiral out of control. Advanced ICT has the potential to provide service platforms able to improve health care. However, dramatic changes to health care provisioning and care models are needed.

The REACTION project [24] aims to address new care models for diabetes management through various clinical applications, such as monitoring of vital signs, feedback provision to the point of care, integrative risk assessment, event, and alarm handling. In addition, the project plans to integrate clinical and organizational workflows with external health information systems for attaining improvements in continuous blood glucose monitoring and tight/safe glycemic control [25]. The envisaged intelligent service platform for management and therapy of diabetes intends to reach different health care regimes across Europe.

### Identifying Advanced Technology Services for Diabetes Management: User Preferences in Europe

User preferences and needs were explored through a series of focus groups conducted in four different European countries [26]. Rather than testing an already developed service within the project, the study explored preferences of end users, who were not involved in the project, about services that are in development phase. Also, the study offered an expanded view of technology management of diabetes that involves individualized care and cultural differences.

The focus groups examined what diabetic patients, nurses, doctors as well as health care professionals, and informal carers would expect from technology, in addition to identifying values, beliefs, hopes, concerns, and needs related to the use of telemonitoring services. Focus groups also highlighted how the use of information technology could potentially change the experience of living with diabetes. Understanding societal factors is a core prerequisite for addressing ethical and social issues at the design stage of technology development.

The focus groups were held in Greece, Italy, Cyprus, and France, with a range of participants including doctors, nurses, social scientists, technical personnel, patients, carers, nutritionists, and lawyers. The questions that guided the discussions focused on several topics including information and risk management of diabetes, security privacy and confidentiality issues, quality of living, monitoring and alert systems, device and sensors design, technical skills, daily activities, and end-users’ concerns. Discussions were informal, encouraging all participants to express their opinion and relate their experiences with diabetes care. Recording devices were not used, and answers have not been attributed to specific participants guaranteeing confidentiality and privacy [27].

Analysis of the focus group discussions identified several topics involved in diabetes and new technologies. The following paragraphs present some of the views and opinions of the participants.

Autonomy and self-management: Technology can assist users to regain a measure of autonomy in managing their condition and prevent long-term risks. Focus group participants viewed technology as a potential personal assistant. An application on a mobile phone could be designed to provide personalized estimation of insulin needs, messages for motivation, and support as well as alerts. However, continuous monitoring may not be essential for improved management of diabetes and could even be seen as information overload. Participants expressed their opinion that technology can improve not only the physical management of the disease but also the social and psychological conditions. However, they also believe that widespread availability of technology at an affordable cost and effective integration into the national health systems remain open issues.

Privacy and confidentiality: Focus group participants expressed their willingness to disclose all information relevant to their condition to their physician, without particular attention to how this information is transmitted. However, if they had the option, they would like all transactions to include strong security mechanisms for any transmitted information. Participants would like to be informed about who is accessing their data, to discern whether their information is shared with trustworthy persons or with someone who may abuse their data. Participants with diabetes appeared less concerned about trust in the use of an Internet platform in conveying personal medical information. However, they were concerned about downtime of devices, inaccuracy of advice, and loss of personal information. Finally, participants were willing to allow their data to be used anonymously for research purposes.

Diabetic person–care provider relationship: All participants expressed the conviction that diabetes management benefits from a close relationship between the person with diabetes and the health care provider team including the physician, nurses, nutritionist, psychology and others. Participants felt that technology may allow for a more accurate, faster response to crisis, as well as better overall management and prevention of complications. However, some participants felt that a careful balance between information and communication is important to avoid information overload and excess. Technology for diabetes management may bring profound changes in self-care and empowerment, as well as in-depth communication with the health providers.

Health management: Management of diabetes requires specific information in predefined time intervals that coupled with personalized algorithms can assist in insulin dosage decisions. A technology platform for diabetes management would require frequent measurements and regular entries of data. Participant willingness for data entry depended on the perceived benefit of the technology services and the time that data entry would require. However, people with diabetes felt that parameters that affect management of their condition, such as emotional and psychological stress, physical exercise, and variability of daily life, are more difficult to define, monitor, predict, and account for. A technology service that could take into consideration some of these parameters would be at a relative advantage compared to a service that uses only glucose measurements and food intake. In addition, participants felt that the amount of information required to be recorded and the ease of use of the technology services will play an important role in the acceptance and use of the services.

## Results

### Closed-Loop and Self-Management of Diabetes Using ICT

State-of-the-art technological solutions have the potential to assist health care professionals, patients, and informal carers to better manage diabetes insulin therapy in a variety of settings, help patients understand their disease, support self-management, and provide a safe environment by monitoring adverse and potentially life-threatening situations with appropriate crisis management.

**Figure 2:**
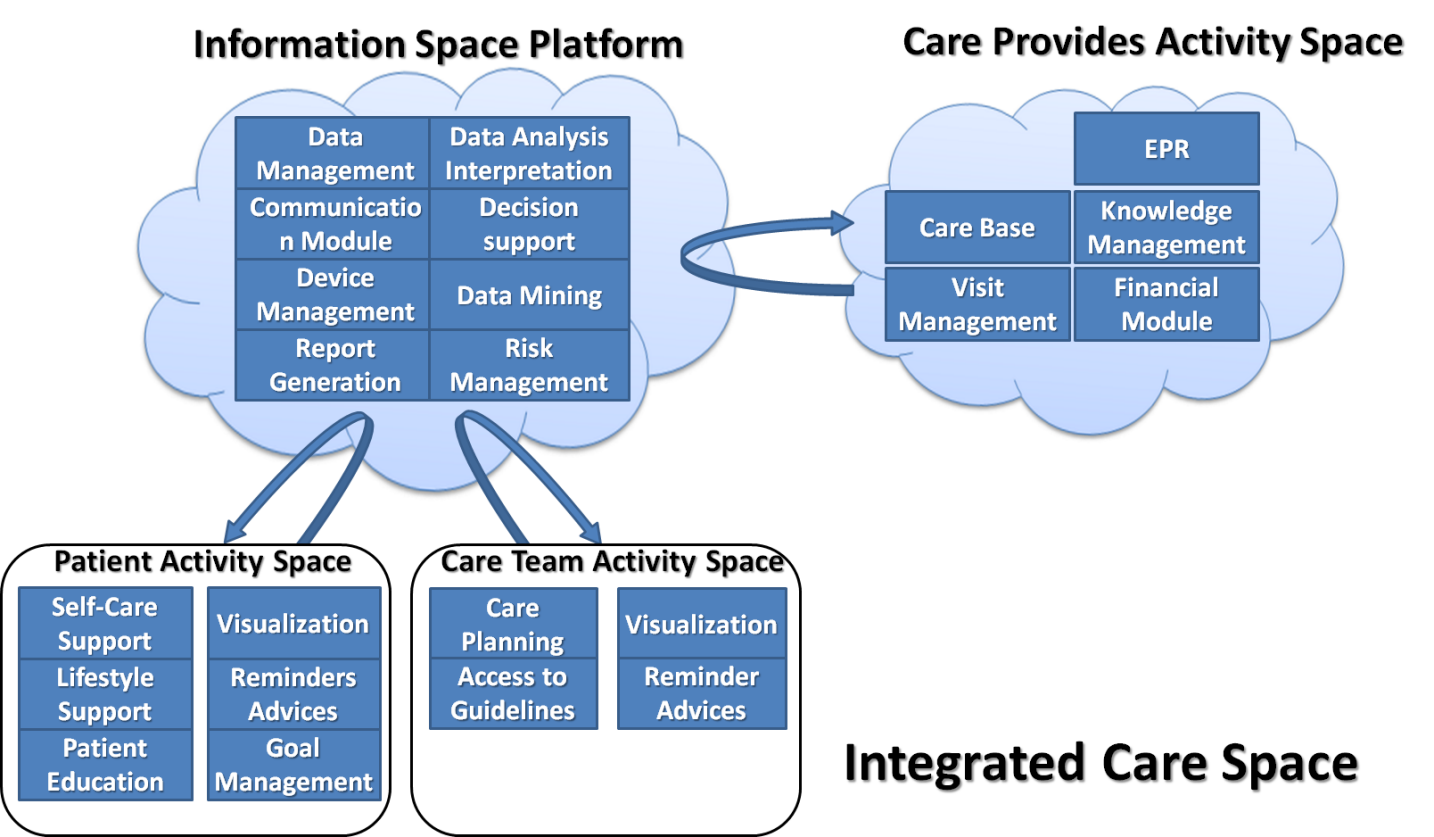
The REACTION platform information and activity spaces and services.

The REACTION project aims to support long-term management of diabetes based on the identified user requirements through modern advanced ICT solutions enabling wearable, continuous blood glucose monitoring, and automated closed-loop delivery of insulin. REACTION is developing a platform that will provide integrated, professional, management and therapy services to diabetes patients in different health care regimes, including professional decision support for in-hospital environments, and safety monitoring for dosage and compliance. This platform provides health care services to diabetes patients and caregivers using new chronic care models that support separation of activity spaces of the information care space (Figure 2) where a closed-loop system is implemented for managing and treating diabetes and for delivering insulin. These include wireless technologies for continuous blood glucose monitoring, clinical monitoring, and intervention strategies, monitoring and predictive risk analysis disease indicators.

The loop has to be closed to health professionals inside hospitals or to the patients (with the main focus on elderly patients in the case of type 2 diabetes) when outside the health care premises. Insulin will be delivered using the appropriate device. In case of automatic glucose management, the loop will be automatically closed on an insulin pump. At such purpose, the availability of glucose sensors with proper accuracy is requested for an effective and efficient functioning of the overall system.

Three clinical field trials are foreseen in the context of the REACTION project: Safe Glycemic Control (SGC) [25] in the hospital ward; chronic care and lifestyle management in the primary care sector; and Automatic Glycemic Control with closed-loop feedback.

While moving towards the full implementation of the platform, three major areas of research and development have been identified and consequently approached: the first one is related to the glucose sensor technology and wearability, the second is related to the platform architecture, and the third to the implementation of the end-user services.

### Evolution in Glucose Sensor Technology and Wearability

The standard of care for measuring glucose levels is by “finger-stick” blood glucose meters. For these, a drop of blood, usually drawn by piercing the skin of a finger, is brought in contact with a test strip. A chemical reaction, commonly mediated by glucose oxidase, glucose dehydrogenase or hexokinase enzymes, triggers an electrochemical sensor or a color reaction that is detected in a reader. The drawback of this method is that only a few measurements can be performed in the course of a day.

At this moment, there are no sensors commercially available that continuously measure blood glucose. Only a few commercially available sensors that allow continuous glucose monitoring in the interstitial fluid, which is a surrogate for blood glucose, have been approved for use. These sensors rely on electrochemical detection of an enzymatic reaction and are minimally invasive. A range of other sensor technologies are currently being tested for their suitability for glucose monitoring. The most promising technologies for continuous glucose monitoring can broadly be classified as follows: enzymatic (electrochemical), impedance spectroscopy / dielectric spectroscopy, optical in the infrared (IR) or near-infrared (NIR) range (IR/NIR absorption, mid-IR emission), polarimetric (eg, anterior chamber of the eye), refractive index, Raman spectroscopy (inelastic photon scattering), photoacoustic (pulsed light absorption dependent on glucose concentration), and others. Moreover, alternatives for invasive sampling are being investigated, but despite significant efforts these technologies are still in a development or evaluation phase and have yet to prove their reliability and accuracy.

**Figure 3:**
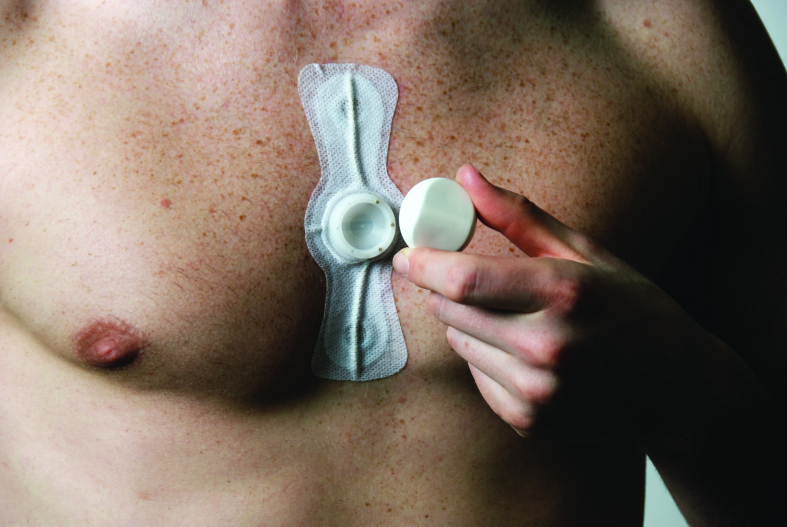
Wearable health monitoring ePatch system from DELTA.

Wearability for sensors for some time can help in the collection of the required measurements. For this purpose, the use of the ePatch technology has been considered (Figure 3) within the REACTION project. The ePatch sensor is a small body sensor, optimized for wearability and bio-compatibility, which senses physiological signals and is embedded in a skin-friendly adhesive. It can contain various types of miniaturized body sensors to measure physiological parameters (eg, ECG, while other vital signs are under development within the context of the REACTION project), microelectronics for data analysis, a wireless radio module for communication, and a battery power source. The basis for the adhesive will be hydrocolloid pressure-sensitive adhesives.

### Platform Architecture

**Figure 4:**
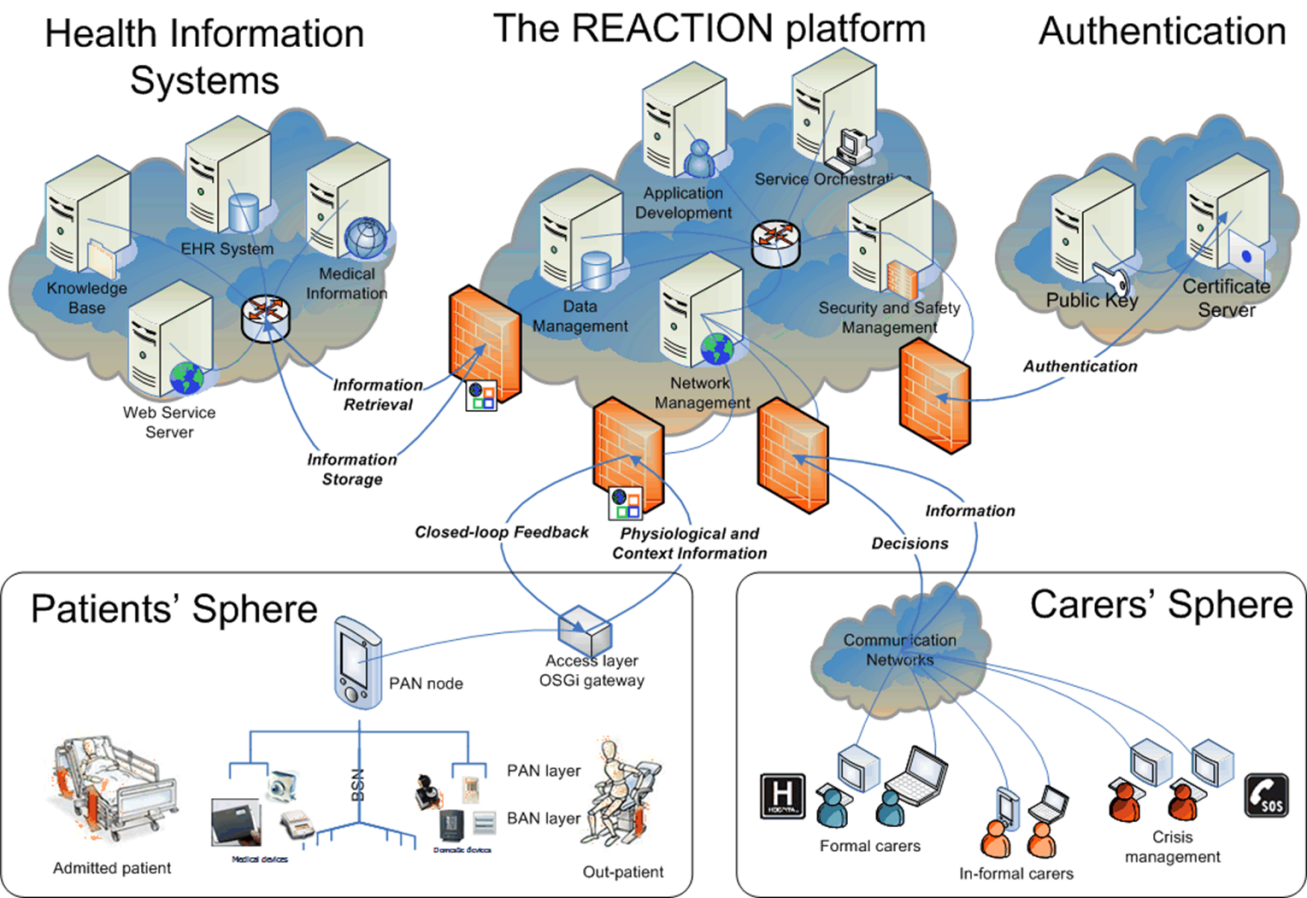
REACTION Platform architecture.

The REACTION platform (Figure 4) is a Service-Oriented Architecture (SOA) platform based on numerous individual services that can be developed and deployed to perform clinical monitoring, execute distributed decision support and security tasks, support workflow management, provide clinical feedback, and perform event handling and crisis management [28]. This approach aims to offer interoperable and loosely coupled services distributed in the network. In this context, a service is a function that is well defined, self-contained, and does not necessarily depend on the context or state of other services.

The REACTION platform, which builds on the LinkSmart [29] middleware for Internet of Things applications, represents each device as web services that can be invoked and consumed by other devices, services, or applications through an overlying peer-to-peer network.

The REACTION platform features a Device Connectivity Kit that enables integration and communication with IEEE 11073 device specializations and other medical devices based on proprietary protocols. It exposes the observations as HL7 v2.6 message format, which are transmitted from the patient sphere to a server where a Rule Engine executes a set of monitoring rules. The rules are expressed by clinicians using a graphical user interface. The platform allows monitoring of vital signs for thresholds as well as long-term trends. An Orchestration Manager allows combining and executing sequences of services and specifying actions such as sending alerts or rising alarms.

### In-hospital Glucose Management System in Compliance With the Medical Device Directive (MDD)

In the first phase of the REACTION project, the implementation of the end-user services has been considered for the hospital ward. A user-centered design approach has been chosen for the development of the first clinical application to be deployed in the endocrinology ward at the Medical University of Graz [30]. The following standards have been considered as relevant for the development of the appropriate software components for REACTION that fall within the scope of the MDD: ISO 13485, IEC 62304, ISO 14971, IEC 62366, and IEC 60601-1-6 [31].

Paper and software mock-ups have been used as a trigger in order to have a basis for discussion and testing. The design process has been supported by regular risk analysis sessions with all involved stakeholders. Derived risks have been collected and incorporated as change requests into the elicited requirements summarized in an extensive specification document. A detailed discussion of the design phase can be found in [32].

Due to maintainability and expandability requirements, it was decided to distinguish between an Android-based user interface and a platform-independent backend, which contains business logic for the electronic decision support system, as well as the data storage and interfaces to the hospital information system. The exchange of data between the backend and frontend components requires mutual authentication and is completely done via encrypted web services to provide data security. The behavior of the frontend application relates to the clinical workflow, which was identified together with end users in the design phase. A requirement issue tracker and task management tool for the documentation of each implementation step was adopted, for supporting the software life cycle processes (IEC 62304 standard), and ensuring an overview of open and already completed requirements, development tasks, and identified bugs.

In addition to the need for detailed documentation, IEC 62304 and IEC 62366 also demand verification and validation during the software life cycle. To verify the functionality after finishing implementation of each system unit, the correct behavior of the application has been tested with simulated user interactions. Already in the early phase of the requirement elicitation process, clinicians stated they would prefer a software system that offers only the required basic functionalities, but with an easy-to-use interface, tailored to the current workflow. In order to avoid poor usability and consequently fulfill the requirements according to IEC 62366, the physicians and nurses have been included in the design of the user interface based on the established clinical workflow, using paper mock-ups and functional prototypes. However, each ward in each hospital usually follows its own workflow; therefore, great attention has been given to the maintainability of the user interface.

**Figure 5:**
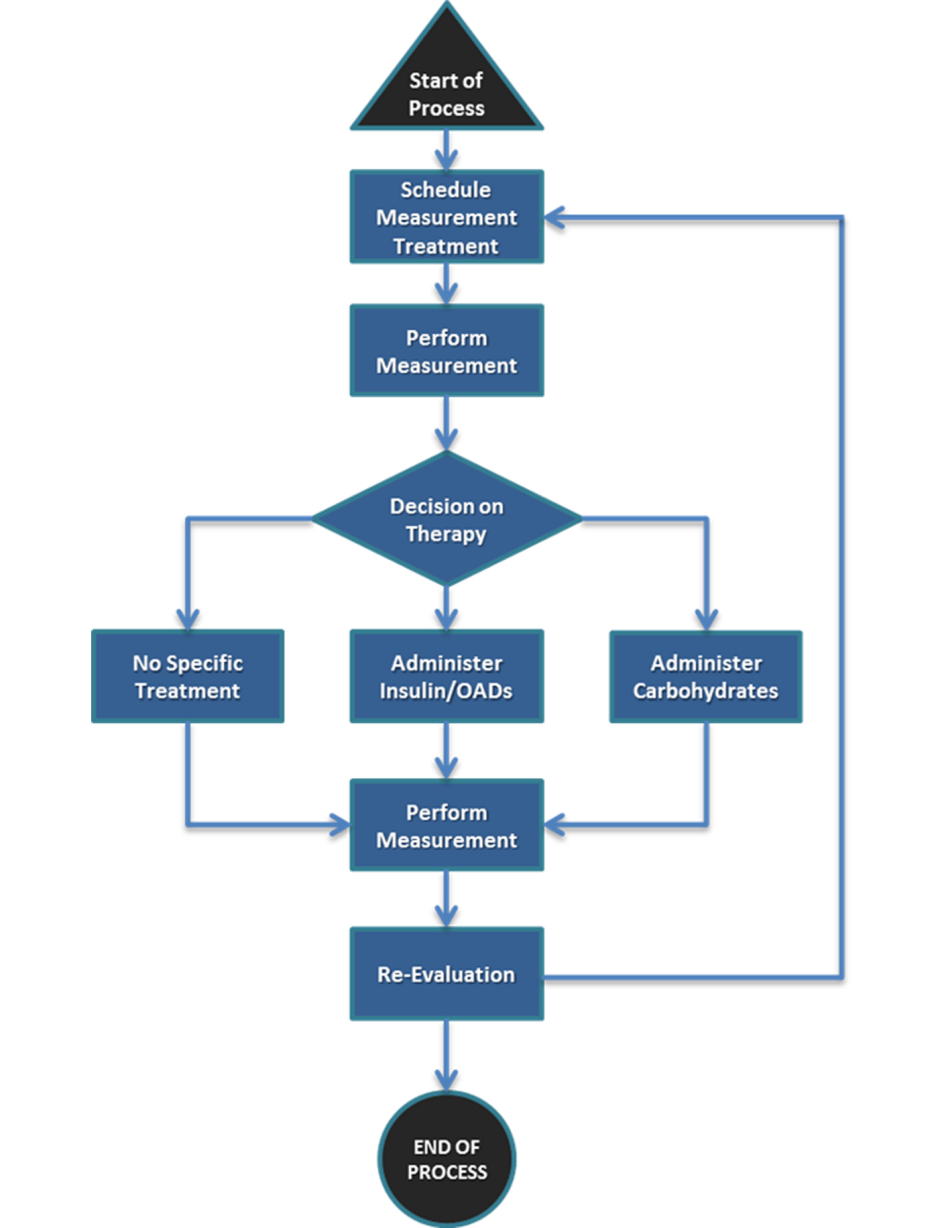
Workflow description of glycemic management of a general ward.

The glucose management workflow system, created as a result of this process, offers decision support for insulin dosing to professional caregivers on a mobile tablet platform that fulfills the need of the users and is compliant with the MDD requirements. This workflow is embedded in the daily routine of the patients, the nursing, and the medical staff. The routine of the latter two groups is very structured and standardized, whereas the patients’ workflows differs from day to day depending on the patients’ health status as well as the planned examinations and their potential delays. These circumstances have to be taken into account for safe glycemic control. The main in-hospital workflow related to glycemic management is shown in Figure 5.

**Figure 6:**
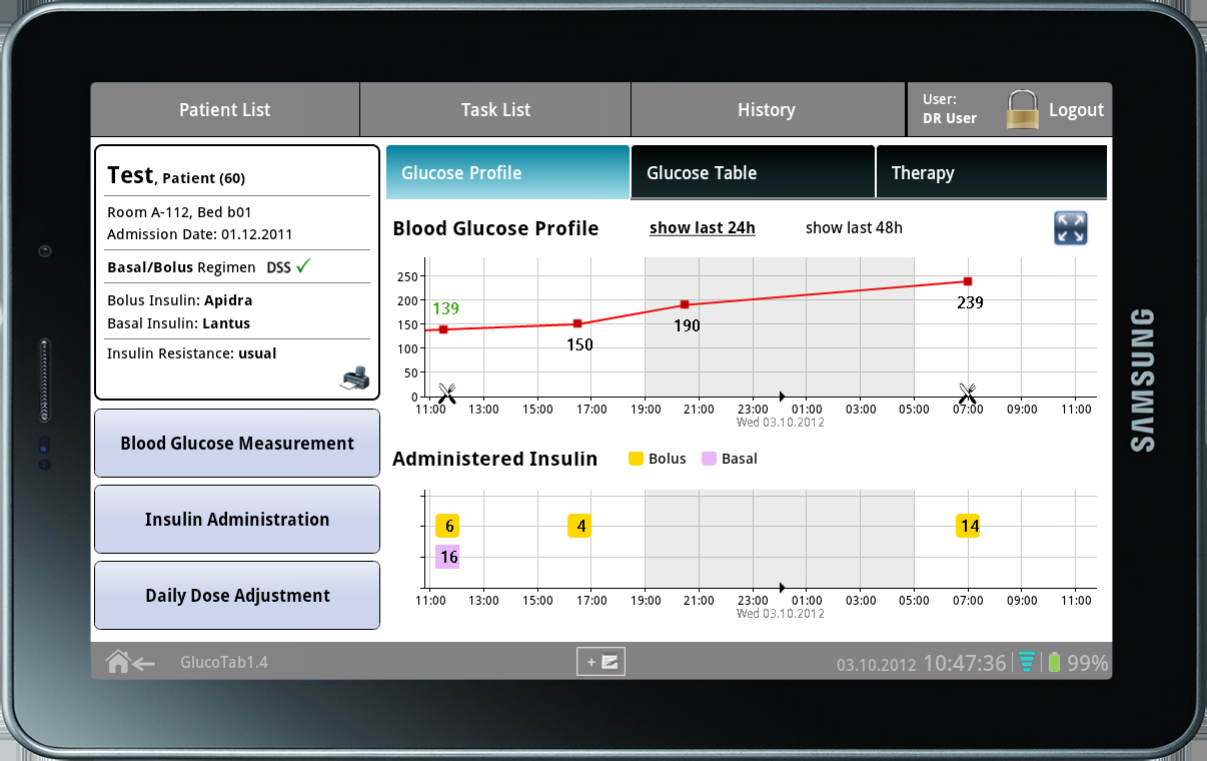
Safe Glycemic Control decision support system.

The Glucose Management System application, developed within the REACTION project (Figure 6), is able to monitor and take into account a range of parameters from various sources including glucose level, nutritional intake, administered drugs, and the patient’s insulin sensitivity. The data are contextualized and algorithms are used to calculate the required insulin doses for SGC. Results are presented to physicians and nurses at the point of care.

This system will be used, according to the REACTION work plan, for the first field trial, involving hospitalized patients with diabetes for the length of their hospital stay, in order to evaluate the feasibility and safety of the automated workflow and insulin dosing support in the ward for endocrinology of the Medical University of Graz.

## Discussion

The trends in care model evolution are influenced by many factors such as biomedical and clinical R&D, financial incentives, technology development, and the socioeconomic environment. An important aspect of future chronic disease management is that personalization, inclusion, and empowerment of the patient have to be essential parts of the care model.

There is abundant evidence, for future diabetes management and therapy, that safe control of the blood glucose level is vital for good diabetes management and insulin therapy. Good glucose control requires frequent measurement of blood glucose levels and complicated algorithms for assessing the insulin dose needed to adjust for short-term variations in activity, diet, and stress. Good control of diabetes, as well as increased emphasis on control of lifestyle factors, may reduce the risk profile of most complications and contribute to health improvement overtime.

The REACTION project aims to respond to these challenges by providing integrated, professional, management and therapy services to diabetes patients in different health care regimes across Europe in an interoperable communication platform.
